# Comparative study of bone regeneration using fibrin sealant with xenograft in rabbit sinus: pilot study

**DOI:** 10.1186/s40902-021-00290-x

**Published:** 2021-02-10

**Authors:** Won-Hyuk Choi, Yong-Deok Kim, Jae-Min Song, Sang-Hun Shin

**Affiliations:** 1 Department of Oral and maxillofacial surgery, School of dentistry, Pusan National University, Yangsan, 50612 Republic of Korea; 2 Department of Oral and maxillofacial surgery, Pusan National University Hospital, Pusan, 49241 Republic of Korea; 3 Dental and Life Science Institute & Dental Research Institute, Pusan National University Dental Hospital, Yangsan, 50612 Republic of Korea; 4 Biomedical Research Institute, Pusan National University Hospital, Pusan, 49241 Republic of Korea

**Keywords:** Animal models, Xenograft, Bone graft, Maxillary sinus, Fibrin sealant

## Abstract

**Background:**

Stability of the grafted bone volume is one of the important factors to the success of alveolar bone grafts. For this, platelet-rich plasma (PRP) or fibrin sealant is mixed with the bone graft material. Bio-Oss® is a protein-free bovine mineral commonly used in bone graft procedures. The grafting particles are commonly combined with a standard fibrin sealant (Tisseel®) to fabricate a plastic implantable product. The purpose of this experiment was to evaluate the efficacy of fibrin sealant (Tisseel®) in bone regeneration performance in a rabbit maxillary sinus model.

**Methods:**

A total of five 3.5 kg weight New Zealand white rabbits were used for the study. After elevating the sinus membrane in both maxillary sinus cavities, Bio-Oss® mixed with normal saline (group 1) was filled into the right side, and Tisseel® mixed Bio-Oss® (group 2) was inserted into the other side. The bone mineral density and bone volume were analyzed with microscopic computed tomography (micro-CT) and histomorphometric 12 weeks after application.

**Results:**

Histologically, new bone formation rate was 14.8%, and grafted bone rate was 70.5% in group 1. In group 2, they were 18.5% and 60.4%, respectively. According to micro-CT analysis, bone mineral density (mg/cm^3^, BMD) was 2.5% larger in group 1.

**Conclusions:**

The findings from this study suggest that, although the difference in the bone formation between group 1 and group 2 appears to be insignificant, group 2 had an advantage in using smaller amount of bone substances to achieve the reliable bone formation.

## Background

In the edentulous state of maxilla, especially posterior area, bone grafting of the maxillary sinus is a critical process for implantation [1]. Numerous maxillary sinus-lifting methods have been reported to manage severe atrophies in the maxillary posterior area [2–5]. One of the critical problems that surgeons face is to prepare efficient grafting materials for the cavities under lifted sinus membrane. Bio-Oss® (Geistlich Biomaterials, Wolhusen, Switzerland) is a protein-free bovine bone product very often used for lifting sinus [3, 6–8]. The materials can be mixed with normal saline or generalized fibrin (Tisseel®) to fabricate plastic materials. The fibrin sealant helps prevent Bio-Oss® particles from scattering. This then ultimately will require less grafting materials to maintain the spaces under lifted sinuses. However, controversies on efficacy of fibrin still exists saying that graft materials with or without Tisseel® yield the similar performance in new bone formation [9–14]. Some studies have shown that adding fibrin to the graft material does not have any positive effects on bone formation [15–20]. These controversial opinions about the efficacy of fibrin materials have provoked a comparative study on the bone regeneration using normal saline or Tisseel®. The purpose of this animal study was to compare the histomorphological and histological findings of protein-free bovine mineral mixed with normal saline and Tisseel® in a rabbit maxillary sinus model.

## Methods

### Animal models

Five New Zealand white rabbits (3.5 kg) were used in this study. The protocol was approved by the Institutional Animal Care and Use Committee.

### Surgical procedure

**Fig. 1:**
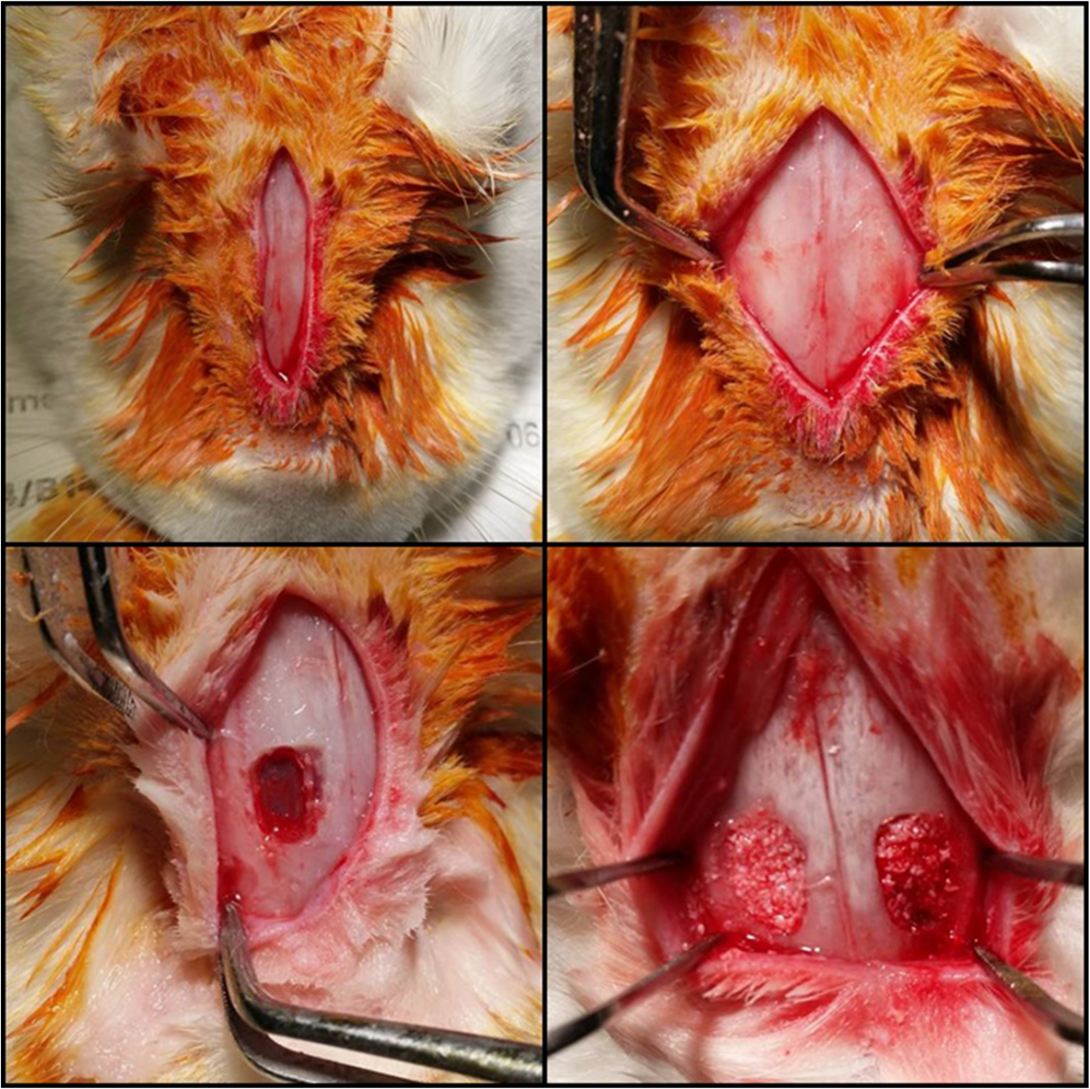
Clinical photographs of surgical procedures. G 1, group 1, Bio-Oss®; G 2, group 2, Bio-Oss® mixed with Tisseel®

All surgical procedures were performed under general anesthesia. A 3-cm vertical incision was given on the median nasal dorsum of rabbit, and the periosteum was elevated. Then, 10-mm^2^ bony window was created in both sinus with a round bur. After lifting the floor of the sinus membrane, the cavities below each sinus membrane was divided into the next two groups according to the materials filled: group 1, Bio-Oss® mixed with normal saline, or group 2, Bio-Oss® mixed with Tisseel^Ⓡ^ (Immuno AG, Vienna, Austria). The normal saline and Tisseel® were mixed with the same volume of Bio-Oss® (Fig. 1).

### Sample preparation and histomorphometry

Animals were sacrificed 12 weeks after the surgery, and bone blocks were excised. Resected bone specimens were fixed in 10% buffered formalin and embedded in methyl-methacrylate resin. The blocks were cut longitudinally through the middle plane of the implants. Histological sections (40 mm) were prepared using a cutting-grinding method and were stained with Masson’s trichrome stain and hematoxylin and eosin stain. The bone mineral density and bone volume were analyzed with micro-CT and histomorphometrics.

The histomorphometry was analyzed by the same inspector using an image analysis system that measures the proportion of newly deposited bone. The system consists of an infinitely calibrated optical microscope, a high-resolution digital camera, an image capture device, and a computer-based image processor for measuring tissue morphology. The Aperio Technologies Scanscope CS system is useful for calculating new bone formation areas on HE-stained slides. The calculation, involving just the drawing of the newly formed bone outlines, is easily done. To calculate the new bone formation area, 3 sites were randomly selected for each slide, the photographs of which were 1 mm × 1 mm. Images of newly formed bones were identified by the color given in each image. They are digitized and sent to a computer for image processing, and a formula is used to analyze the quantity percentage of the total area of ​the defect:

$$ \mathrm{A}\ \mathrm{new}\ \mathrm{bone}\ \mathrm{formation}\ \mathrm{ratio}\ \left(\%\right)=\frac{\mathrm{New}\kern0.5em \mathrm{bone}\kern0.5em \mathrm{in}\kern0.5em \mathrm{defect}\kern0.5em \mathrm{site}\kern0.5em }{\mathrm{Total}\kern0.5em \mathrm{defect}\kern0.5em \mathrm{site}}\times 100 $$

## Results

### Gross observations

**Fig. 2:**
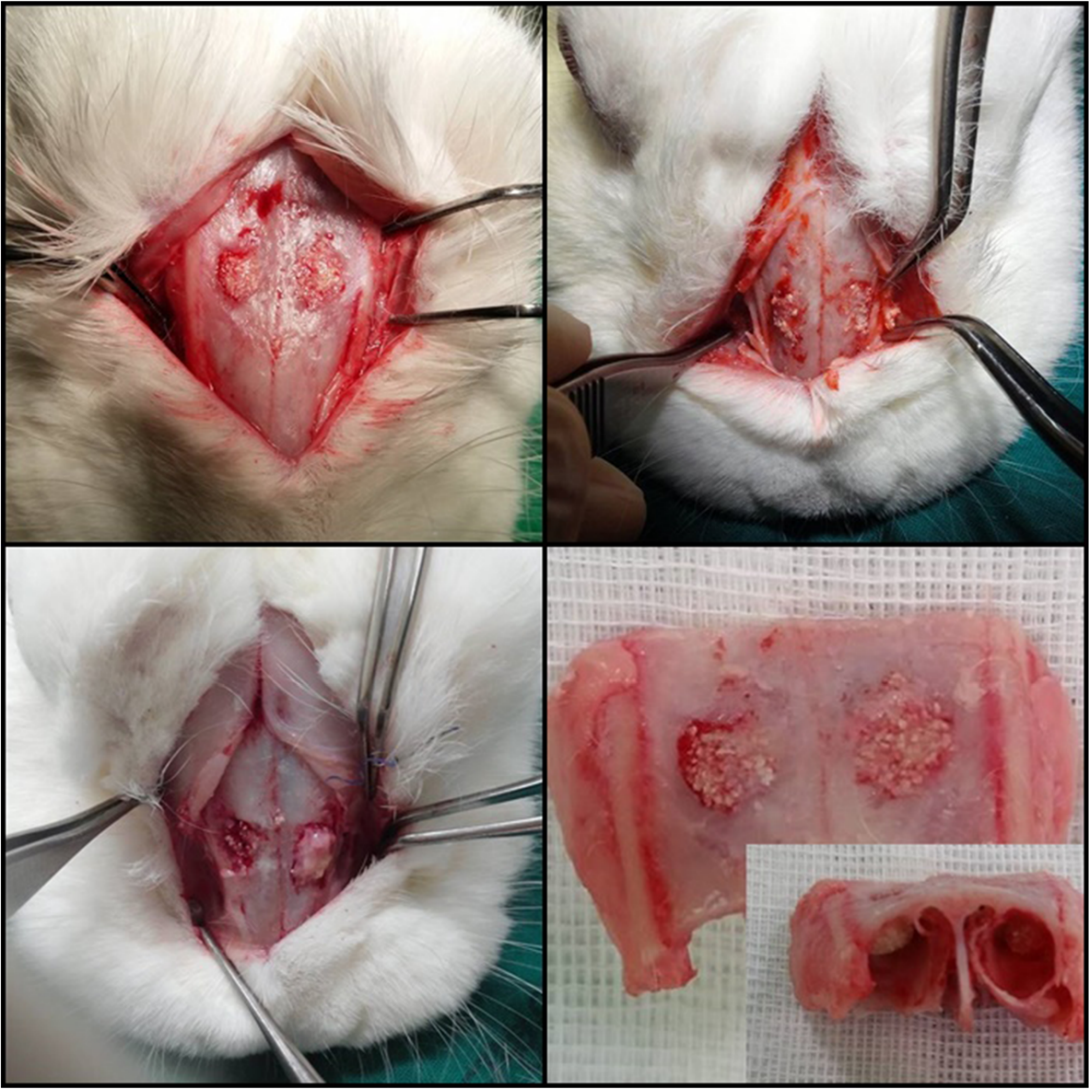
Photograph of experiment after sampling. G 1, group 1, Bio-Oss®; G 2, group 2, Bio-Oss® mixed with Tisseel®

All animals recovered from surgery and healed without any complications until the end of the experiment. During the operation, animals showed no clinical signs of sinusitis. Post-operative healing was within normal range in all cases (Fig. 2).

### Histologic and histomophometric findings

**Fig. 3:**
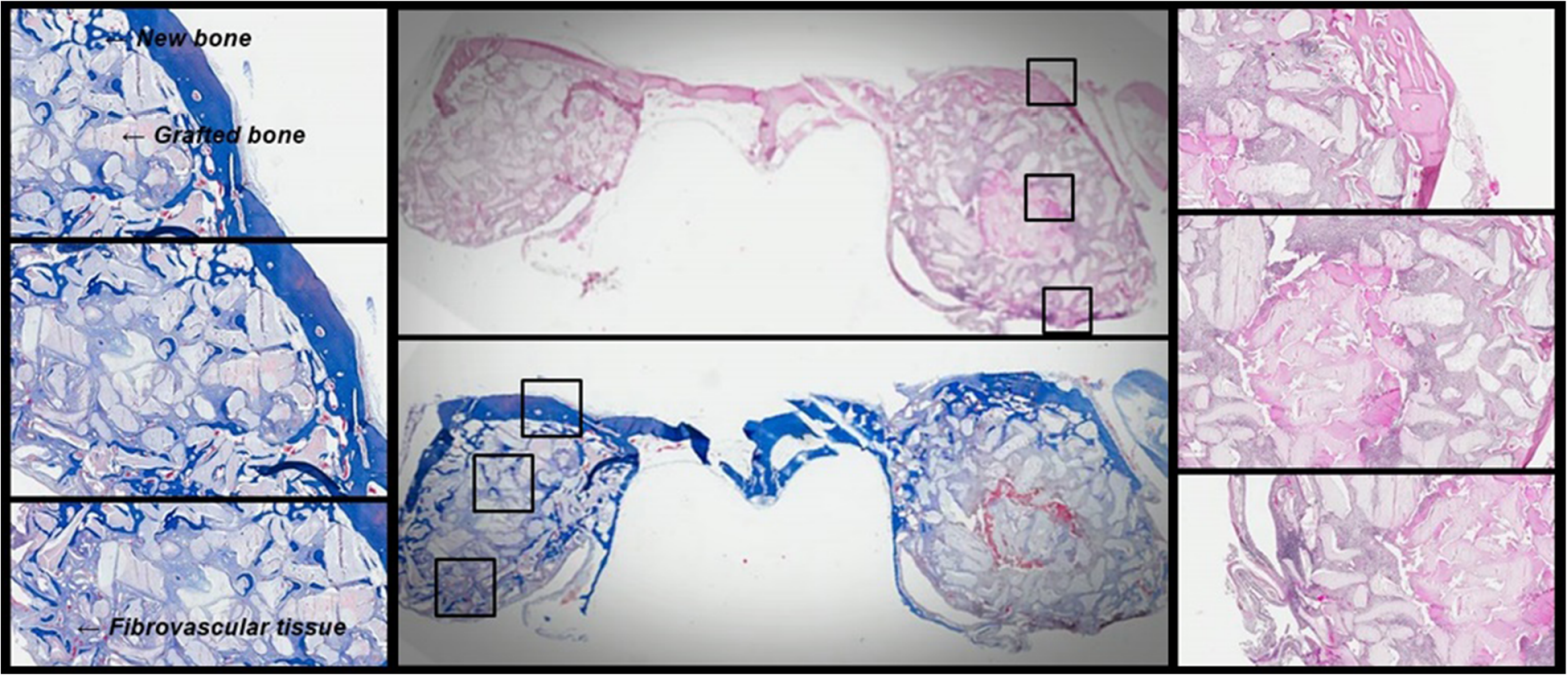
Photomicrograph shows grafted region after 12 weeks. **a** Masson’s trichrome stain × 100. **b** Overall view. **c** Hematoxylin and eosin stain × 100. G 1, group 1, Bio-Oss®; G 2, group 2, Bio-Oss® mixed with Tisseel®

**Fig. 4:**
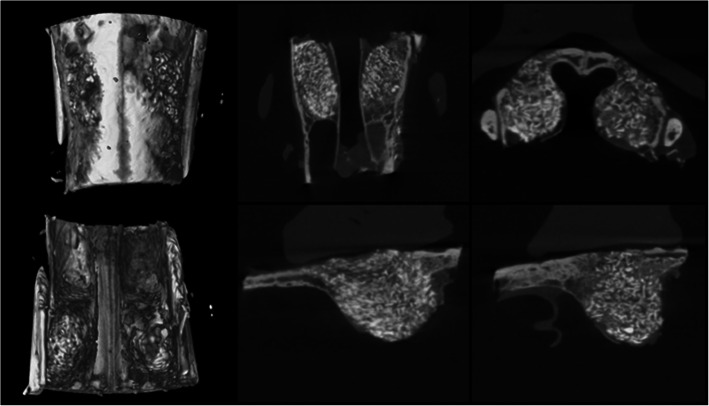
Micro-CT analysis. **a** Three-dimensional image. **b** Axial view. **c** Coronal view. **d** Right sagittal view. **e** Left sagittal view. G 1, group 1, Bio-Oss®; G 2, group 2, Bio-Oss® mixed with Tisseel®

Twelve weeks after the sinus lift, an examination of the bone specimens showed that in the Tisseel® mixed Bio-Oss® site the grafted particles were surrounded by a layer of newly formed bone (Fig. 3). The new bone formation rate was 18.5%. The new bone surrounding the grafted particles was also surrounded by fibrovascular tissue (Fig. 3). In the normal saline mixed Bio-Oss® site, the grafted particles were also surrounded by new bone but smaller amounts of new bone (Fig. 3). The new bone formation rate was 14.8%. In the contrary, the amount of grafted bone rate was larger in the normal saline mixed Bio-Oss® site compared to the Tisseel® mixed Bio-Oss® site (70.5% vs. 60.4%) (Table 1). In the composition bone volume measurement through micro-CT analysis (Fig. 4), group 2 showed that the amount of graft used was smaller than group 1 (Table 2).

**Table 1:** Histologic analysis of groups 1 and 2

	Group 1			Group 2		
	NB	GB	FT	NB	GB	FT
No. 1	0.15 ± 0.04	0.68 ± 0.04	0.17 ± 0.03	0.18 ± 0.04	0.59 ± 0.03	0.21 ± 0.06
No. 2	0.17 ± 0.02	0.74 ± 0.04	0.11 ± 0.02	0.18 ± 0.05	0.60 ± 0.06	0.16 ± 0.03
No. 3	0.13 ± 0.03	0.70 ± 0.06	0.15 ± 0.07	0.20 ± 0.04	0.62 ± 0.04	0.24 ± 0.04
No. 4	0.14 ± 0.03	0.70 ± 0.05	0.14 ± 0.07	0.15 ± 0.05	0.61 ± 0.08	0.22 ± 0.03
No. 5	0.15 ± 0.03	0.70 ± 0.03	0.16 ± 0.04	0.22 ± 0.04	0.60 ± 0.04	0.22 ± 0.08
Mean ± SD	0.15 ± 0.04	0.70 ± 0.01^*^	0.15 ± 0.02^*^	0.19 ± 0.02	0.60 ± 0.01^*^	0.21 ± 0.03^*^

*NB* new bone, *GB* grafted bone, *FT* fibrovascular tissue

Group 1 Bio-Oss®, group 2 Bio-Oss® mixed with Tisseel®

^*^P < 0.05

**Table 2:** Volume measurements in groups 1 and 2 using micro-CT

	Group 1			Group 2		
	TV (mm^3^)	BV (mm^3^)	BV/TV (%)	TV (mm^3^)	BV (mm^3^)	BV/TV (%)
No. 1	33.84	32.31	95.48	43.27	34.60	79.96
No. 2	87.67	82.81	94.46	89.01	70.26	78.93
No. 3	48.67	47.15	96.88	56.09	45.55	81.21
No. 4	52.59	51.27	97.49	36.06	29.03	80.50
No. 5	88.61	87.09	98.29	77.12	64.21	83.26
Mean ± SD	62.27 ± 24.62	60.12 ± 23.78	96.51 ± 1.54^*^	60.31 ± 22.37	48.73 ± 18.03	80.77 ± 1.62^*^

*TV* total augmented volume, *BV* volume of bone

Group 1 Bio-Oss®, group 2 Bio-Oss® mixed with Tisseel®

^*^P < 0.05

## Discussion

Sinus-lifting surgery is a routinely performed technique for increasing the height of the pneumatized posterior maxilla. Sinus floor augmentation is considered a highly successful procedure, but complications during or after surgery can still occur [21, 22]. The most frequent surgical complication is perforation of Schneiderian membrane, with reported incidence between 10 and 55% [23, 24]. Schneiderian membrane at the base of this upper jaw may be perforated for anatomical reasons, surgical risk factors, or pathophysiological factors [25]. The underside of the membranes is a unique space where graft materials are placed between hard and soft tissues, and constant respiratory air pressure exists, making it difficult to maintain volume stability. The perforation of membrane which can occur during surgery or postoperatively is a risk factor that can cause postoperative acute and chronic sinusitis due to particulate materials. To prevent this, the grafting material should be confined to the space underneath the membrane. Fabrication of grafting bone particles may involve PRP that are collected from the patient’s blood or Tisseel®, a commercial product. Drawing blood samples to collect PRP can cause discomfort to the patient and thus it can negatively affect rapport between the surgeon and a patient.

The results indicate that the incorporation of fibrin sealant with the bone graft materials showed insignificant differences. The rate of new bone formation was slightly higher than control groups, indicating that the materials needed for the lifting reduced in amount. In group 1, the bone density measured was high. In the case of fibrovascular tissue, it was higher in group 2.

In the case of Bio-Oss® mixed with Tisseel® for maxillary sinus lift, bone formation in group 2 was higher than group 1. Group 2 showed some signs of inflammation. This may have caused Tisseel® to impair the initial vascularization of the biomaterial and consequently limited the bone growth to the implant site. The reason for this may be due to the accumulation of granulation tissue with decomposing collagen tissue on the heavily damaged site. The results obtained from this study are similar to those from previously conducted animal experiments. Carmagnola et al. showed that the integration ratio between Bio-Oss® and bone surface was significantly higher in defects treated with Bio-Oss® mixed with Tisseel® [15, 16]. Luht et al. studied bone formation and local blood flow over a graft containing fibrin sealant mixed with iliac bone in standardized defects in dog’s tibia [26]. This study indicated that the fibrin sealant did not work to encourage the formation of new bones and fibrous capsules formed around grafted particles.

On the other hand, the grafted bone volume was smaller in using Tisseel® mixed site. This means that even if only a small amount of graft material is used in the group using Tisseel®, the success rate of bone transplantation is the same as that of the site where normal saline was mixed and transplanted. It was found that the difference in BMD by our micro-CT analysis was not significantly different from the case of using normal saline. The volume of the implant has to do with the time when new bones are formed. The time taken for the formation of new bone is affected by the amount of grafted bone. Consequently, the amount of biomaterial required for a defect should be carefully measured and decided. The fact that the mixed compounds have a positive effect on the handling and adhesion of skeletal defect walls has been pointed out before [27].

These physical and biological properties are obviously helpful for surgeons. For this reason, the results of this study suggest that Tisseel® can serve as a delivery system for grafting particles in the sinus floor elevation, although there are clinical limitations such as the time of melting in use and relatively high cost.

## Conclusions

This experiment was a pilot experiment to examine the advantages and disadvantages of the use of Tisseel^Ⓡ^ when the maxillary sinus elevation was performed using Bio-Oss^Ⓡ^ mixed with normal saline and Tisseel^Ⓡ^. Through this experiment, it can be speculated that the use of Tisseel^Ⓡ^ will reduce the amount of bone to be transplanted, and as a result, the bone healing period for dental implant placement after surgery will ultimately be reduced.

## Data Availability

Not applicable.

## Abbreviations

PRP: Platelet-rich plasma
micro-CT: Microscopic computed tomography
BMD: Bone mineral density
